# Path Planning Algorithm for Unmanned Surface Vessel Based on Multiobjective Reinforcement Learning

**DOI:** 10.1155/2023/2146314

**Published:** 2023-02-15

**Authors:** Caipei Yang, Yingqi Zhao, Xuan Cai, Wei Wei, Xingxing Feng, Kaibo Zhou

**Affiliations:** ^1^MOE Key Laboratory of Image Information Processing and Intelligent Control, School of Artificial Intelligence and Automation, Huazhong University of Science and Technology, Wuhan 430074, China; ^2^Wuhan Second Ship Design and Research Institute, Wuhan 430205, China

## Abstract

It is challenging to perform path planning tasks in complex marine environments as the unmanned surface vessel approaches the goal while avoiding obstacles. However, the conflict between the two subtarget tasks of obstacle avoidance and goal approaching makes the path planning difficult. Thus, a path planning method for unmanned surface vessel based on multiobjective reinforcement learning is proposed under the complex environment with high randomness and multiple dynamic obstacles. Firstly, the path planning scene is set as the main scene, and the two subtarget scenes including obstacle avoidance and goal approaching are divided from it. The action selection strategy in each subtarget scene is trained through the double deep *Q*-network with prioritized experience replay. A multiobjective reinforcement learning framework based on ensemble learning is further designed for policy integration in the main scene. Finally, by selecting the strategy from subtarget scenes in the designed framework, an optimized action selection strategy is trained and used for the action decision of the agent in the main scene. Compared with traditional value-based reinforcement learning methods, the proposed method achieves a 93% success rate in path planning in simulation scenes. Furthermore, the average length of the paths planned by the proposed method is 3.28% and 1.97% shorter than that of PER-DDQN and dueling DQN, respectively.

## 1. Introduction

In ocean exploration, the competition among countries to protect marine territorial sovereignty and develop marine resources has become increasingly fierce. The unmanned surface vessel (USV), as a kind of vessel with high autonomy, has broad application prospects in the field of ocean exploration. As one of the current research hotspots, the path planning of USV faces many challenges, including unknown environment, perceptual uncertainty, and dynamic obstacles [1–3]. The USV path planning is aimed to obtain a collision-free path under specific circumstances. It can be divided into two subtarget tasks, such as goal approaching and obstacle avoidance. The goal approaching method helps the USV reach the destination, focusing on reducing path length and travel time. The obstacle avoidance method makes the USV conduct real-time collision avoidance through a series of decisions [4].

Traditional path planning methods perform well in simple known static environments and reach a destination while avoiding obstacles [5–9]. But there are still major deficiencies in the exploration and decision-making capabilities of algorithms in complex environments, failing to guarantee the success rate and environmental adaptability. Currently, the deep reinforcement learning (DRL) methods have advantages in unknown environment exploration and real-time action decision making in path planning problems [10, 11]. Therefore, the use of DRL methods to solve the path planning problem has become one of the new research directions [12]. For example, Tai et al. used radar observations and target positions as inputs and applied DRL methods to path planning tasks for the first time [13]. The agent uses the discrete control commands generated by the algorithm to avoid obstacles in the indoor mobile environment. Chen et al. proposed an intelligent collision avoidance algorithm with DRL improving the path quality compared with optimal reciprocal collision avoidance (ORCA) [14]. Chen et al. constructed the interaction model between the agent and the obstacle, providing the basis for the reinforcement learning strategy of the agent's path planning in complex dynamic environment [15]. Thus, it is effective to use DRL algorithms for goal approaching and dynamic obstacle avoidance.

However, the path length inevitably increases in the obstacle avoidance process, which conflicts with the requirement of destination reaching for the goal approaching subtarget task. Therefore, it is difficult for a single optimization strategy to simultaneously achieve these subtarget tasks. Recently, intelligence computing algorithms have been widely used in related fields [16–19]. A more comprehensive model can be obtained in ensemble learning by combining multiple weak learners [20]. Inspired by the idea of integrated learning, a multiobjective reinforcement learning architecture is designed to trade off these subtarget tasks. There is a need to investigate the USV path planning based on multiobjective reinforcement learning.

Main contributions in this paper can be summarized as follows:

1. Based on the main scene of path planning considering random goals and multiple dynamic obstacles, the dynamic obstacle avoidance subtarget scene and the goal approaching subtarget scene are constructed. The double deep *Q*-network with prioritized experience replay (PER-DDQN) is applied to the action decision of USV in two scenes, respectively.
2. A multiobjective reinforcement learning architecture based on ensemble learning is designed, optimizing the multiobjective policy integration method in the USV path planning task.
3. A USV path planning algorithm based on multiobjective reinforcement learning is proposed, improving the success rate of USV path planning tasks and shortening the planned path length in the complex environment.

The rest of this paper is organized as follows. The theoretical background of the PER-DDQN and the multiobjective reinforcement learning method is introduced in Section 2. The proposed algorithm is introduced in Section 3. Simulation experiments and results are presented in Section 4. Discussion is given in Section 5, and Section 6 concludes this paper.

## 2. Related Work

### 2.1. *Q*-Learning

The *Q*-learning algorithm is a value-based reinforcement learning algorithm [21]. A *Q*-value table is built and updated in the *Q*-learning algorithm. Each action is selected with the greatest benefit based on the *Q*-value. The maximum *Q*-value of the next state is used to estimate the *Q*-value of the current state. The update formula is as follows:

(1) $$\begin{array}{c} Q\left(s,a\right)=Q\left(s,a\right)+\alpha \left[r+\gamma \;\max_{a^{\prime }}\;Q\left(s,a\right)-Q\left(s,a\right)\right]\text{,} \end{array}$$

where *Q*(*s*, *a*) denote agent's expectation of reward for performing action *a* in state *s*. *α* represents the learning rate and *γ* represents the discount factor. The reward obtained by the agent after performing action a is *r*, and the state is changed to *s*′. *Q*(*s*, *a*) denote agent's expectation of reward for performing action *a*′ in state *s*′.

### 2.2. Deep *Q*-Network

To address the curse of dimensionality in high-dimensional state spaces, Mnih et al. used a neural network with *θ* to approximate the Q-value: *Q*(*s*, *a*; *θ*) ≈ *Q*(*s*, *a*) [22]. DQNs are optimized by reducing and minimizing *L*_*i*_(*θ*_*i*_)=*E*_*s*,*a*,*r*,*s*′_[(*y*_*i*_^DQN^ − *Q*(*s*, *a*; *θ*_*i*_))^2^] at each iteration *i*, with target *y*_*i*_^*DQN*^=*r*+*γ* max_*a*′_ *Q*(*s*, *a*; *θ*_*i*_^−^). Here, *θ*_*i*_^−^ are the parameters of a target network that is frozen for a number of iterations while updating the online network *Q*(*s*, *a*; *θ*_*i*_) by gradient descent. The action a is chosen from *Q*(*s*, *a*; *θ*_*i*_) by an action selector, which typically implements an *ε*-greedy policy that selects the action that maximizes the *Q*-value with a probability of 1-*ε* and chooses randomly with a probability of *ε*.

### 2.3. Experience Replay

Online reinforcement learning (RL) agents incrementally update their parameters (of the policy, value function or model) while they observe a stream of experience [23]. Because the agent discards experience after one update in simple reinforcement learning, rare valid experience is underutilized. At the same time, there is a substantial correlation between neighbouring experiences, which is not favourable to model training. By storing experiences in replay memory, experience replay can effectively solve the above problems. It becomes possible to break the temporal correlations by mixing more and less recent experience for the updates [24].

### 2.4. Related Literature

Value function-based DRL algorithm uses deep neural network to approximate value function or action value function and uses temporal difference or *Q*-learning, respectively, to update the value function or action value function. Many scholars use DRL methods based on value functions, including DQN algorithm and some improved variant algorithms, to motivate robots or other agents to obtain optimal paths [25–27]. Additionally, with the introduction of the strategy gradient method, DRL based on strategy gradient is used in robot path planning, such as A3C [28], DDPG [29], TRPO [30], and PPO [31]. When it comes to agent data control and management, blockchain hyperledger fabric is one of the practical technologies [32, 33]. We have briefly summarized some of the recent literature, as shown in Table 1.

## 3. Methodology

When the USV performs a mission in a complicated marine environment with various dynamic impediments, it needs to arrive at its destination without colliding with the obstacles. It is necessary to create a model that can select appropriate actions in different states in order to achieve dynamic obstacle avoidance and goal approaching.

### 3.1. PER-DDQN

**Figure 1:**
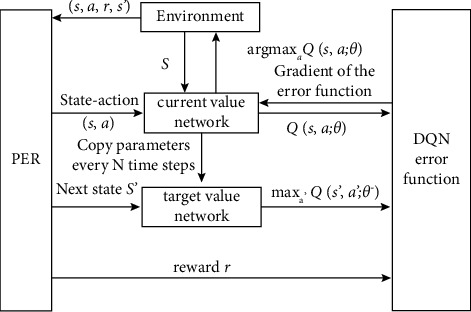
Strategy iteration and optimization based on PER-DDQN reinforcement learning algorithm.

The PER-DDQN improves the learning effect and the learning speed by introducing the DDQN and priority experience replay. Two *Q*-networks are used in DDQN to eliminate the bias caused by the greedy policy [34]. The current *Q*-network is used to calculate the action corresponding to the maximum *Q*-value, and the target *Q*-network is used to calculate the target *Q*-value corresponding to the maximum action. Prioritized experience replay is a stochastic sampling method that interpolates between pure greedy prioritization and uniform random sampling [35]. The probability of being sampled is monotonic in a transition's priority, while guaranteeing a nonzero probability even for the lowest priority transition. The probability of sampling transition *i* is defined as

(2) $$\begin{array}{c} P\left(i\right)=\frac{p_{i}^{\alpha }}{\sum_{k}p_{k}^{\alpha }}\text{,} \end{array}$$

where *i* is the priority of transition. The exponent *α* determines how much prioritization is used, with *α* = 0 corresponding to the uniform case. In the actual process, all samples can be divided into *n* intervals, and uniform sampling is performed in each interval. The PER-DDQN is used for the action decision of the agent in the constructed scene. The flowchart of the algorithm is shown in Figure 1.

### 3.2. Framework for Multiobjective Reinforcement Learning

The path planning task of the USV includes two subtarget tasks, such as dynamic obstacle avoidance and goal approaching. The traditional reinforcement learning architecture for a single task is no longer appropriate. A multiobjective reinforcement learning architecture is built for policy learning and ensemble in the main scene of path planning, inspired by ensemble learning.

**Figure 2:**
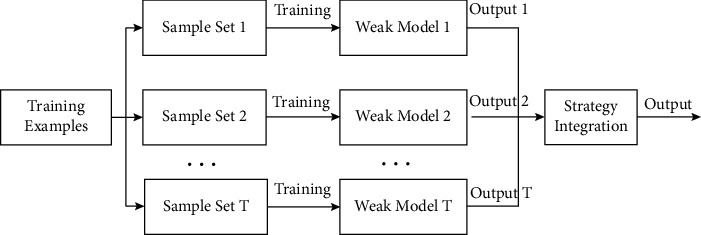
Flowchart of the bagging algorithm.

The fundamental principle of ensemble learning is to integrate the learning results of numerous weak models to produce better overall results, which can be classified into bagging, boosting, and stacking. The sample training set is sampled with replacement in the bagging method, yielding *T* independent sample sampling sets. *T* weak learners are trained from *T* sample sets. Weighted average, voting, and other strategy integration approaches are employed to provide final decision results [36].  Figure 2 depicts the flowchart.

**Figure 3:**
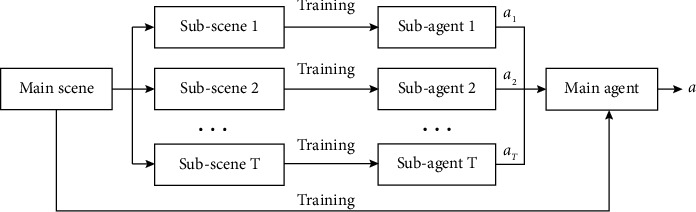
Proposed framework for multiobjective reinforcement learning.

Corresponding to weak learners in ensemble learning, the designed multiobjective reinforcement learning architecture leverages subagents for training in subtarget scenes. Different from traditional integration methods, the proposed method uses a main agent based on the reinforcement learning method for policy integration. According to the environmental state of the main scene, the main agent selects the strategy of the subagent in the corresponding state of the subscene and makes a decision. The designed multiobjective reinforcement learning architecture is shown in Figure 3.

### 3.3. The Proposed Approach

**Figure 4:**
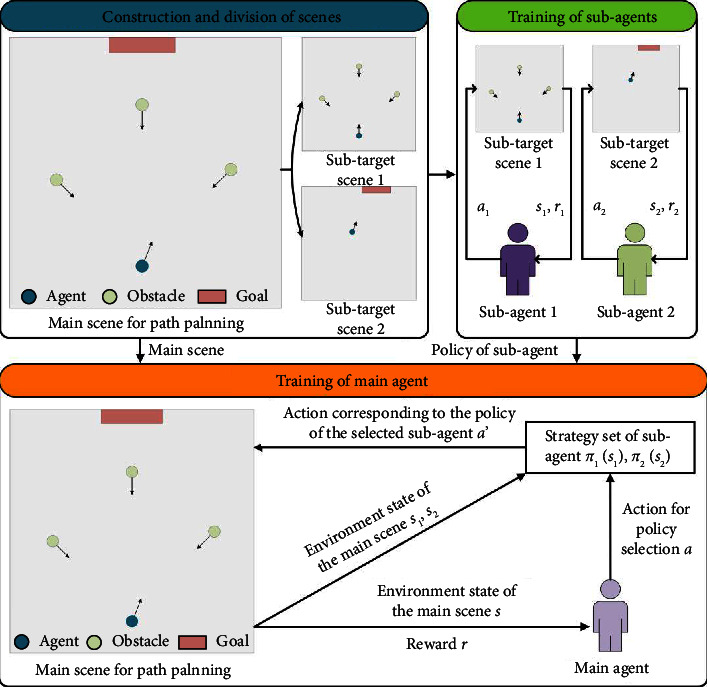
The overall process of the path planning algorithm for USV based on multiobjective reinforcement learning.

The PER-DDQN algorithm is combined with the designed architecture for the constructed path planning scene, and a USV path planning algorithm based on multiobjective reinforcement learning is proposed.  Figure 4 depicts the overall process of the proposed method.

 
*Step 1*. The subagents in each subtarget scene are trained using the PER-DDQN algorithm, and the strategies of each subagent are saved.
 
*Step 2*. In the constructed path planning main scene, the main agent is trained by the PER-DDQN method. The main agent selects subagent according to the current environment state and gives the actions according to the strategy of the selected subagent in this state.
 
*Step 3*. The main agent executes actions of the selected subagent, generating and storing experience for the main agent to learn from.

## 4. Simulation Experiments

The main scene, dynamic obstacle avoidance subtarget scene, and goal approaching subtarget scene are built in Unity3D to verify the effectiveness of the proposed method. The settings for scenario conditions and reinforcement learning parameters are provided separately. Algorithms were written by Python 3.8 and processed by a server with a RAM (64G) and a CPU (Intel Core i9-11900K).

### 4.1. Scene Building

**Figure 5:**
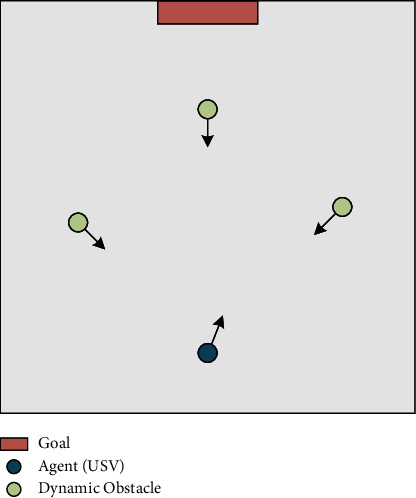
Main scene for USV path planning.

The main scene of path planning considering random goal and multiple dynamic obstacles is generated on a two-dimensional plane, as illustrated in Figure 5, to represent the complicated marine environment.

**Figure 6:**
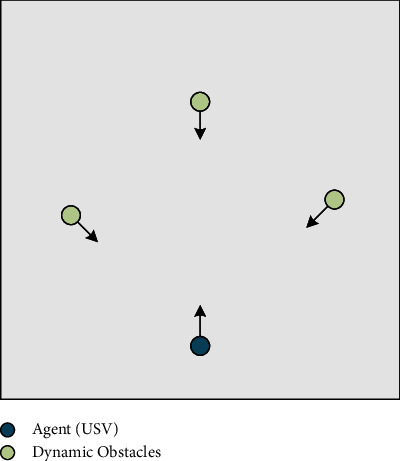
Dynamic obstacle avoidance subtarget scene.

The dynamic obstacle avoidance subtarget scene is built on the basis of the main scene, as shown in Figure 6, to focus on the dynamic obstacle avoidance subtarget. The agent does not need to consider the problem of goal approaching and instead attempts to travel through the obstacle region without colliding. It is deemed effective obstacle avoidance when the agent's ordinate is larger than the ordinate of all obstacles. When a collision occurs, it is regarded as obstacle avoidance failure.

**Figure 7:**
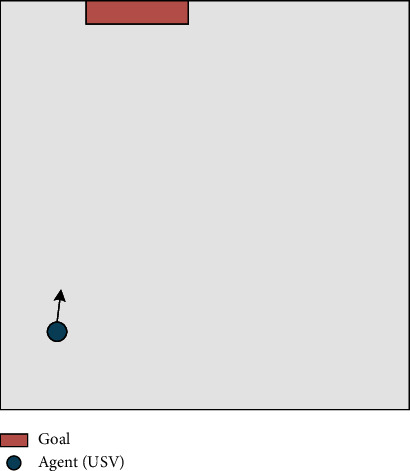
Goal approaching subtarget scene.

The goal approaching subtarget scene is built on the basis of the main scene, as shown in Figure 7, to focus on the goal approaching subtarget. Dynamic obstacles are removed, and the only learning objective is to approach the goal.

### 4.2. Simulation Setup

#### 4.2.1. Initial Conditions

The initial conditions of agents, dynamic obstacles, and goals in the main scene and each subtarget scene (dynamic obstacle avoidance and goal approaching) are set to random values to ensure that the training model generalizes and meets the actual application requirements.

*(1) Dynamic Obstacle Avoidance Subtarget Scene*. For dynamic obstacles, set its radius to 0.5 m, the maximum speed to 1 m/s, and the quantity to 3. The states of three obstacles are set using the *A*∗ path planning algorithm and the ORCA dynamic obstacle avoidance algorithm to avoid mutual collision. The coordinates of dynamic obstacles' starting points are randomly selected in square areas centered on (0 m, 6 m), (5 m, 5 m), and (−5 m, 5 m), respectively. Also, the coordinates of dynamic obstacles' end points are randomly selected in square areas centered on (0 m, −6 m), (−5 m, −5 m), and (5 m, 5 m). The area of each area is 4 m^2^. For the agent, set its radius to 0.5 m and the maximum speed to 1 m/s. The agent's initial abscissa and ordinate are chosen randomly from the range [−2 m, 2 m], [−4 m, −8 m].

*(2) Goal Approaching Subtarget Scene*. The goal is a rectangle with a length of 5 m and a width of 1 m. The initial abscissa of the target is randomly selected within the range of [−5 m, 5 m]. The initial abscissa and ordinate of the agent are chosen randomly from the range [−6 m, 6 m], [−8 m, 4 m].

*(3) Main Scene*. The initial condition of the goal is consistent with the goal in the goal approaching subtarget scene, and the motion parameters of the dynamic obstacle are consistent with dynamic obstacles in the dynamic obstacle avoidance subtarget scene. The agent's initial abscissa is chosen randomly from the range [−2 m, 2 m], [−4 m, −8 m], and the initial ordinate is set to −8 m.

#### 4.2.2. Reinforcement Learning Parameter

The reinforcement learning settings for the agent in each scene, such as the action space, state, and rewards, are set as follows.

**Figure 8:**
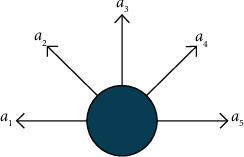
Action space of agent.

*(1) Action Space Setting*. The agent's action space is set to 5 directions divided evenly into in the main scene and each subscene, as shown in Figure 8, to reduce the training cost.

*(2) State Settings*. The states of the agent (*s*_1,*t*_ and *s*_2,*t*_) are set as equations (3) and (4) in two subtarget scenes (dynamic obstacle avoidance and goal approaching):

(3) $$\begin{array}{c} s_{1,t}=\left(s_{self1},s_{obs1},s_{obs2},s_{obs3}\right)\text{,} \end{array}$$

(4) $$\begin{array}{c} s_{2,t}=\left(s_{self2},s_{tgt}\right)\text{,} \end{array}$$

where *S*_*self*1_ and *S*_*self*2_ are the states of the agent in two subtarget scenes, represented by agent's positions, respectively, at *t* − 2, *t* − 1, *t*. *S*_*obs*1_, *S*_*obs*2_, and *S*_*obs*3_ are the states of the obstacles in dynamic obstacle avoidance subtarget scene, represented by obstacles' positions, respectively, at *t* − 2, *t* − 1, *t*. *S*_*tgt*_ is the state of the goal in goal approaching subtarget scene, represented by goal's position, respectively, at *t*.

The state of agent *s*_*t*_ is set as equation (5) in the main scene:

(5) $$\begin{array}{c} s_{t}=\left(s_{self}^{\prime },s_{obs1}^{\prime },s_{obs2}^{\prime },s_{obs3}^{\prime },s_{tgt}^{\prime }\right)\text{,} \end{array}$$

where *s*_obs1_′, *s*_obs2_′, *s*_obs3_′, and *s*_self_′ are the states of the obstacles and the agent in the main scene, represented by their positions, respectively, at *t* − 2, *t* − 1, *t*. *s*_*tgt*_′ is the state of the goal in the main scene, represented by goal's position, respectively, at *t*. In dynamic obstacle avoidance subtarget scene, the dimensions of action space and observation space are 5 and 8, respectively. In goal approaching subtarget scene, the dimensions of action space and observation space are 5 and 3, respectively. In dynamic obstacle avoidance subtarget scene, the dimensions of action space and observation space are 5 and 9, respectively.

*(3) Reward Setting*. The rewards of the agent (*r*_1,*t*_, *r*_2,*t*_, and *r*_*t*_) are set as equations (6)–(8) in two subtarget scenes and the main scene:

(6) $$\begin{array}{c} r_{1,t}=\left\{\begin{array}{cc} -0.5 & if:1<obs\_\;di\;st<1.05\text{,}\\ -2 & obs\_\;di\;st=1\text{,}\\ 1.5 & y_{t}>y_{i,t},\forall i=1,2,3\text{,}\\ 0.3*\left(y_{t}-y_{t-1}\right)-0.015 & else\text{,} \end{array}\right. \end{array}$$

(7) $$\begin{array}{c} r_{2,t}=\left\{\begin{array}{cc} 1.5 & if:{target}_{collide\;d}\text{,}\\ 0.2*\left(pre\_\;di\;st\right)-0.005 & else\text{,} \end{array}\right. \end{array}$$

(8) $$\begin{array}{c} r_{t}=\left\{\begin{array}{cc} -0.5 & if:1<obs\_\;di\;st<1.05\text{,}\\ -1.5 & obs\_\;di\;st=1\text{,}\\ 1.5 & y_{t}>y_{i,t},\forall i=1,2,3\text{,}\\ 0.5*\left(pre\_\;di\;st-di\;st\right) & -0.005\text{,} \end{array}\right. \end{array}$$

where *obs*_*dist* represents the distance between the agent and the dynamic obstacle at time *t*. *y*_*t*_ and *y*_*t−*1_ are the ordinates of the agent at time *t* and time *t* − 1, respectively. *target_collided* indicates whether the agent is in contact with the goal, and *dist* and *pre_dist* are the distances between the agent and the goal at time *t* and time *t* − 1, respectively. The training parameters of the reinforcement learning algorithm in each scene are shown in Table 2.

### 4.3. Result Analysis

**Figure 9:**
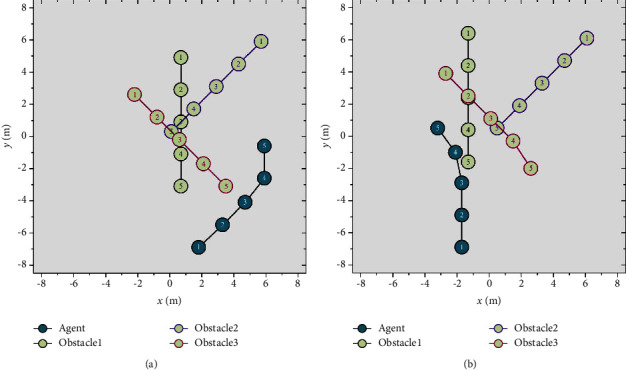
Experimental results of the agent's dynamic obstacle avoidance subscene.

After 800 times of training in the dynamic obstacle avoidance subtarget scene, the two samples are shown in Figure 9. The agent's obstacle avoidance strategy is slightly different in different scenes. The dynamic obstacles are evenly dispersed in front of the agent, as indicated in Figure 9(a), and the collision risk is substantial. The agent chooses to move to the right, avoiding the range where obstacles might congregate. The dynamic obstacles are concentrated at the agent's front right, as shown in Figure 9(b), and the agent chooses to go straight at the start. When there is a risk of collision, the agent turns left to avoid obstacles urgently.

**Figure 10:**
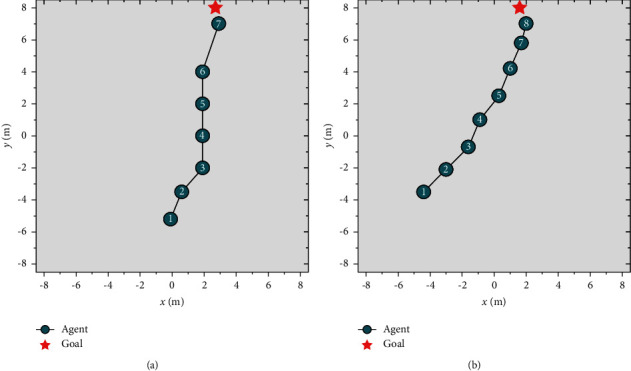
Experimental results of the agent's target approach subscene.

After 800 times of training in the goal approaching subtarget scene, the two samples are shown in Figure 10. When the goal is in front of the agent, as shown in Figure 10(a), the agent continues to adjust at the beginning and end of the path while moving forward in the middle. When the goal is far from the front of the agent, as shown in Figure 10(b), the agent remains adjusted throughout. The results show that the agent can rapidly approach the goal under various initial conditions without the interference of dynamic obstacles.

**Figure 11:**
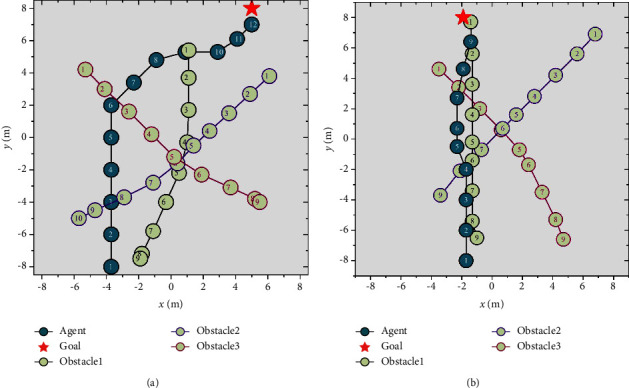
Experiment results of the main scene of agent path planning.

After 800 times of training in the main scene, the two samples are shown in Figure 11. As shown in Figure 11(a), the obstacles are distributed in front of the agent. At the same time, the target is far from the front of the agent. The agent chooses to move sideways quickly after going straight through the obstacle area in the initial stage to approach the goal. As shown in Figure 11(b), the obstacles are evenly distributed in front of the agent. At the same time, the target is near the front of the agent. The agent chooses to go straight and dynamically avoid collision in the obstacle area.

The experimental results show that by dynamically selecting the strategy of subagents, the main agent can avoid obstacles and approach the goal in various scenes to accomplish the path planning task well. Therefore, the effectiveness of the proposed method has been verified.

## 5. Discussion

To verify the effectiveness of the proposed framework on strategy integration, a comparison is made between reinforcement learning methods that use integration methods such as linear voting method and rank voting method and our method. At the same time, the proposed method is compared with *A*∗ + ORCA and the path planning algorithm based on single-objective reinforcement learning to demonstrate the advantages of the proposed method in path planning tasks.

### 5.1. Comparison with Other Ensemble Learning Algorithms

In the linear voting method, the *Q*-value of each action in the main scene is the normalized sum of the *Q*-values in the corresponding states of each subscene. In the rank voting method, the rank of each action in the main scene is the sum of the ranks of the corresponding states of each subscene. In these methods, the subagents and their strategies are consistent with the proposed method. The performance indicators of these methods in the results of 100 random experiments are shown in Table 3.

The rank voting method has the worst integration effect and the lowest success rate. Compared with the rank voting method, the linear voting method considerably enhances the success rate by keeping the path length from increasing. The path length of the proposed method is slightly longer than that of the other two methods, but the success rate of goal approaching and dynamic obstacles avoidance are higher. The proposed method has the best overall performance.

**Figure 12:**
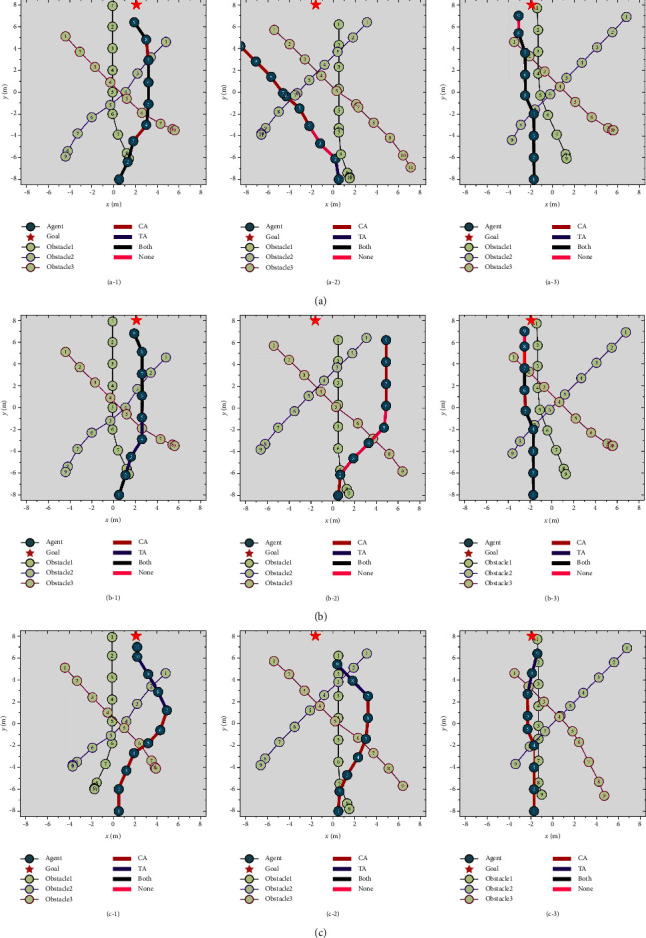
Path planning results of various ensemble learning methods in the same environment: (a) rank voting, (b) linear voting, and (c) proposed method.

Three random samples of the path planning results of the three methods in the same environment are shown in Figure 12. “CA” represents the main agent to choose the strategy in the dynamic obstacle avoidance subtarget scene. “TA” represents the main agent to choose the strategy in the goal approaching subtarget scene. Each agent makes better decision in low-complexity environments to avoid obstacles and approach the goal. In a more complicated context, however, the agents using the traditional ensemble method face the issue of disordered decision making. The decision strategy of the obstacle avoidance agent is selected in the initial stage of the path to maximize the success rate of obstacle avoidance. When approaching the goal, the decision strategy of the goal approaching agent is selected to maximize the success rate of goal approaching. The proposed method has a greater success rate of path planning in varied situations than the other two ensemble methods, demonstrating the superiority of the proposed ensemble learning architecture over the traditional ensemble methods.

### 5.2. Comparison with Other Path Planning Algorithms

The training times of PER-DDQN are 2400 times, and other hyperparameters are consistent with the method's parameter setting in the main scene. The performance indicators of these methods in the results of 100 random experiments are shown in Table 4.

The assumptions of the ORCA in the decision-making process are inconsistent with the requirements of dynamic obstacle avoidance in practical applications. Therefore, the success rate of agent using the *A*^∗^ + ORCA is low. DDQN algorithm solved the problem of overestimation of action value function in *Q*-learning. On this basis, PER-DDQN uses priority sampling to accelerate the convergence speed of the algorithm, and dueling DQN uses the competitive architecture to estimate the value function more precisely. They perform well in the constructed scenes. Our approach combines the strengths of reinforcement learning with ensemble learning. The experimental results show that the method proposed in this paper has the best overall performance when considering path length and success rate.

**Figure 13:**
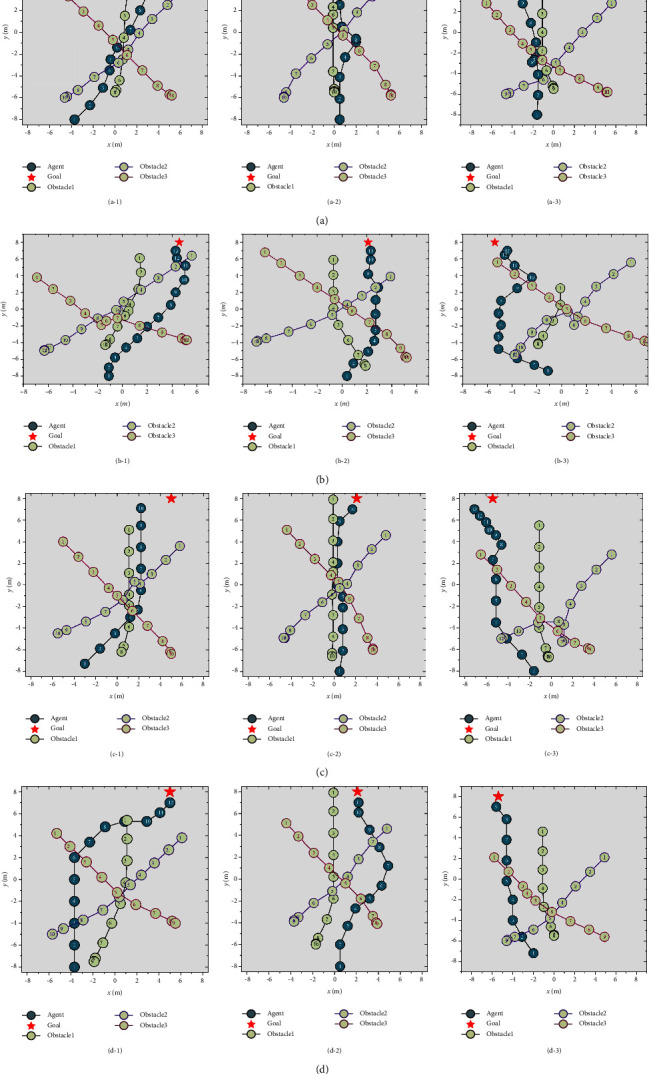
Path planning results of various path planning methods in the same environment: (a) *A*^∗^ + ORCA, (b) dueling DQN, (c) PER-DDQN, and (d) proposed method.

Four random samples of the path planning results of the four methods in the same environment are shown in Figure 13. The policies provided by the *A*^∗^ + ORCA method are not sufficient for the agent to always avoid obstacles. The policies provided by dueling DQN are conservative, and there may be detours. The policies provided by the PER-DDQN are not mature enough in dealing with conflicts between subtarget tasks. Many problems still exist such as long planning path, failure to avoid obstacles, and approaching the goal. The experimental results show that the method proposed in this paper is generally safe and performs well in various environments.

## 6. Conclusion

In this paper, a path planning algorithm for USVs in complex marine environments based on multiobjective reinforcement learning is proposed. To simulate complex ocean environment, a complex scene including dynamic obstacles and random goal is built. On this basis, two subtarget scenes with goal approaching and dynamic obstacle avoidance are established, respectively. The PER-DDQN algorithm is used to train the action decision of the agent in the two subtarget scenes. A multiobjective reinforcement learning architecture is designed to optimize the agent's policy integration method in path planning. The simulation results show that the proposed method achieves a higher path planning success rate and a shorter path length than the traditional path planning methods.

Although the proposed method realizes the decision making of the agents in the constructed scenes, the complexity of the scene is still insufficient. The computational efficiency and path planning success rate of the algorithm will be reduced in complex environments. Modelling more actual scenes and building more realistic training scenes can effectively improve the adaptability of the algorithm. In addition, the action space in the established model is discrete, which is somewhat different from the real world. Agents cannot output continuous action decisions in the scenario of discrete action strategy only. The assumption that the next time step after the action can reach the target position is also idealized, and inertial factors need to be taken into account to optimize the model. In future work, hostile ships with tracking capabilities will be added to the scene to train the model better. The dimension of the action space will be increased to enhance the USV's mobility. In addition, changing the scene from 2-dimensional space to 3-dimensional space is our follow-up research direction.

## Figures and Tables

**Table 1:** Related literature.

	Study title	Approach	Merit	Limitations	Ref
DRL based on value function	An improved algorithm of robot path planning in complex environment based on double DQN	Double DQN	The problem of lacking experiments is solved by redefining the initialization of the robot and the reward function for the free position	Slow convergence speed of the algorithm	[25]
	The USV path planning of dueling DQN algorithm based on tree sampling mechanism	Dueling DQN	The algorithm can identify and avoid static obstacles in the environment and realize autonomous navigation in complex environments	Internal connection between the state-action pairs is not strong enough	[26]
	Tactical UAV path optimization under radar threat using deep reinforcement learning	DQN-PER	Alleviates the sparse reward problem	Overvaluation of the action-state value	[27]

DRL based on strategy gradient	Advanced double layered multi-agent systems based on A3C in real-time path planning	A3C	The correlation between state distribution samples is eliminated, and the sample storage mode of experience playback mechanism is replaced	Convergence to local optimal strategy	[28]
	The path-planning algorithm of unmanned ship based on DDPG	DDPG	The algorithm can be applied to continuous state space and action space	Sensitive to hyperparameters	[29]
	Hindsight trust region policy optimization	TRPO	The algorithm can choose a more appropriate step length during training	Large environments and policies are prone to large errors	[30]
	PPO-based reinforcement learning for UAV navigation in urban environments	PPO	The algorithm has better data efficiency and robustness	The difference between the old and new policies cannot be too large with each update	[31]

**Table 2:** Training parameters for reinforcement learning.

Scene	Learning rate	Batch_size	Discount factor	FC structure
Dynamic obstacle avoidance	0.0005	256	0.99	[64, 64, 32]
Goal approaching	0.001	256	0.99	[32, 32, 16]
Main scene	0.0005	256	0.99	[32, 32, 16]

**Table 3:** Performance of various ensemble learning methods.

Method of policy integration	Success rate of path planning (%)	Success rate of goal approaching (%)	Success rate of dynamic obstacle avoidance (%)	Length of the path (m)
Rank voting	52	55	91	17.68
Linear voting	57	63	94	16.98
Proposed method	93	96	97	17.96

**Table 4:** Performance of various path planning methods.

Method of path planning	Success rate of path planning (%)	Success rate of goal approaching (%)	Success rate of dynamic obstacle avoidance (%)	Length of the path (m)
*A * ^∗^ + ORCA	42	100	42	16.43
Dueling DQN	82	97	85	18.32
PER-DDQN	80	98	81	18.57
Proposed method	93	96	97	17.96

## Data Availability

The data used to support the findings of this study are available from the corresponding author upon request.

## References

[1] Yuh J., Marani G., Blidberg D. R. (2011). Applications of marine robotic vehicles. *Intelligent service robotics*.

[2] Liu X., Li Y., Zhang J., Zheng J., Yang C. (2019). Self-adaptive dynamic obstacle avoidance and path planning for USV under complex maritime environment. *IEEE Access*.

[3] Liu Y., Bucknall R. (2015). Path planning algorithm for unmanned surface vehicle formations in a practical maritime environment. *Ocean Engineering*.

[4] Guo S., Zhang X., Du Y., Zheng Y., Cao Z. (2021). Path planning of coastal ships based on optimized DQN reward function. *Journal of Marine Science and Engineering*.

[5] Singh Y., Sharma S., Sutton R., Hatton D., Khan A. (2018). A constrained A∗ approach towards optimal path planning for an unmanned surface vehicle in a maritime environment containing dynamic obstacles and ocean currents. *Ocean Engineering*.

[6] Gao M. Y., Hu B. B., Liu B. Constrained path-planning control of unmanned surface vessels via ant-colony optimization.

[7] Xia G., Han Z., Zhao B., Wang X. (2020). Local path planning for unmanned surface vehicle collision avoidance based on modified quantum particle swarm optimization. *Complexity*.

[8] Yao Y. l, Liang X. f, Li M. z (2021). Path planning method based on D∗ lite algorithm for unmanned surface vehicles in complex environments. *China Ocean Engineering*.

[9] Liang X., Jiang P., Zhu H. Path planning for unmanned surface vehicle with dubins curve based on GA.

[10] Zhai H., Wang W., Zhang W. Path planning algorithms for USVs via deep reinforcement learning.

[11] Xiong L., Kang Y., Zhang P. (2018). Research on behavior decision-making system for unmanned vehicle[J]. *Automobile Technology*.

[12] Xu H., Wang N., Zhao H., Zheng Z. (2019). Deep reinforcement learning-based path planning of underactuated surface vessels. *Cyber-Physical Systems*.

[13] Tai L., Paolo G., Liu M. Virtual-to-real deep reinforcement learning: continuous control of mobile robots for mapless navigation.

[14] Chen Y. F., Liu M., Everett M. Decentralized non-communicating multiagent collision avoidance with deep reinforcement learning.

[15] Chen C., Liu Y., Kreiss S. Crowd-robot interaction: crowd-aware robot navigation with attention-based deep reinforcement learning.

[16] Hu Z., Zhang Y., Xing Y., Zhao Y., Cao D., Lv C (2022). Toward human-centered automated driving: a novel spatiotemporal vision transformer-enabled head tracker. *IEEE Vehicular Technology Magazine*.

[17] Hu Z., Xing Y., Gu W., Cao D., Lv C. (2023). Driver anomaly quantification for intelligent vehicles: a contrastive learning approach with representation clustering. *IEEE Transactions on Intelligent Vehicles*.

[18] Zhou K., Yang C., Liu J., Xu Q. (2022). Dynamic graph-based feature learning with few edges considering noisy samples for rotating machinery fault diagnosis. *IEEE Transactions on Industrial Electronics*.

[19] Yang C., Liu J., Zhou K., Yuan X., Ge M. F. (2022). Transfer graph-driven rotating machinery diagnosis considering cross-domain relationship construction. *IEEE*.

[20] Dong X., Yu Z., Cao W., Shi Y., Ma Q. (2020). A survey on ensemble learning. *Frontiers of Computer Science*.

[21] Watkins C. J. C. H., Dayan P. (1992). Technical note: Q-learning. *Machine Learning*.

[22] Mnih V., Kavukcuoglu K., Silver D. (2013). Playing atari with deep reinforcement learning. https://arxiv.org/abs/1312.5602.

[23] Schaul T., Quan J., Antonoglou I. (2015). Prioritized experience replay. https://arxiv.org/abs/1511.05952.

[24] Zhang S., Sutton R. S. (2017). A deeper look at experience replay. https://arxiv.org/abs/1712.01275.

[25] Zhang F., Gu C., Yang F. (2022). An improved algorithm of robot path planning in complex environment based on Double DQN. *Advances in Guidance, Navigation and Control*.

[26] Huang Z., Liu S., Zhang G. The USV path planning of Dueling DQN algorithm based on tree sampling mechanism.

[27] Alpdemir M. N. (2022). Tactical UAV path optimization under radar threat using deep reinforcement learning. *Neural Computing & Applications*.

[28] Lee D., Kim J., Cho K., Sung Y. (2021). Advanced double layered multi-agent Systems based on A3C in real-time path planning. *Electronics*.

[29] Xu D., Liu X., Huang Z. (2022). The path-planning algorithm of unmanned ship based on DDPG[J]. *International Core Journal of Engineering*.

[30] Zhang H., Bai S., Lan X. (2019). Hindsight trust region policy optimization. https://arxiv.org/abs/1907.12439#:%7E:text=HTRPO%20leverages%20two%20main%20ideas,to%20select%20conductive%20hindsight%20goals.

[31] Chikhaoui K., Ghazzai H., Massoud Y. PPO-based reinforcement learning for UAV navigation in urban environments.

[32] Khan A. A., Laghari A. A., Gadekallu T. R. (2022). A drone-based data management and optimization using metaheuristic algorithms and blockchain smart contracts in a secure fog environment. *Computers & Electrical Engineering*.

[33] Yang Y., Wang W., Xu R. AoI optimization for UAV-aided MEC networks under channel access attacks: a game theoretic viewpoint.

[34] Zhu Z., Hu C., Zhu C., Zhu Y., Sheng Y. (2021). An improved dueling deep double-Q network based on prioritized experience replay for path planning of unmanned surface vehicles. *Journal of Marine Science and Engineering*.

[35] Zhai P., Zhang Y., Shaobo W. (2022). Intelligent ship collision avoidance algorithm based on DDQN with prioritized experience replay under COLREGs. *Journal of Marine Science and Engineering*.

[36] Breiman L. (1996). Bagging predictors. *Machine Learning*.

